# Incidence and predictors of HBV relapse after cessation of nucleoside analogues in HBeAg-negative patients with HBsAg ≤ 200 IU/mL

**DOI:** 10.1038/s41598-017-02010-w

**Published:** 2017-05-12

**Authors:** Chih-Chien Yao, Chao-Hung Hung, Tsung-Hui Hu, Sheng-Nan Lu, Jing-Hung Wang, Chung-Mo Lee, Chien-Hung Chen

**Affiliations:** Division of Hepatogastroenterology, Department of Internal Medicine, Kaohsiung Chang Gung Memorial Hospital and Chang Gung University College of Medicine, Kaohsiung, Taiwan

## Abstract

The predictors of hepatitis B virus (HBV) relapse and HBsAg loss after cessation of nucleos(t)ide analogues (NA) in HBeAg-negative patients with end-of-treatment HBsAg ≤ 200 IU/mL remains unclear. The study recruited 119 chronic hepatitis B (CHB) patients who achieved end-of-treatment HBsAg ≤ 200 IU/mL, were treated with lamivudine (n = 34) and entecavir (n = 85). The 5-year rates of post-treatment virological relapse, clinical relapse, and HBsAg loss at 60 months were 39.4%, 27.6%, and 45.9%, respectively. Cox regression analysis revealed that HBV DNA at entry and end-of-treatment HBsAg levels were independent predictors of virolgical and clinical relapse. HBV genotype C and end-of-treatment HBsAg were independent factors of HBsAg loss. Patients with a combination of end-of-treatment HBsAg < 50 IU/mL and HBV DNA < 2 × 10^5^ IU/mL at entry experienced the lowest virological and clinical relapse rates (5% and 0% at 60 months, respectively). In contract, patients with a combination of end-of-treatment HBsAg ≥ 50 IU/mL and HBV DNA ≥ 2 × 10^5^ IU/mL at entry experienced high virological and clinical relapse (80.7% and 71.5% at 60 months, respectively). No patients experienced hepatic decompensation when clinical relapse occurred after timely retreatment. A combination of HBV DNA levels at entry and end-of-treatment HBsAg levels was useful for predicting the post-treatment HBV relapse in HBeAg-negative patients with HBsAg ≤ 200 IU/mL.

## Introduction

Chronic hepatitis B (CHB) is a major global health problem^1, 2^ and a leading cause of liver-related morbidity and mortality, especially in Taiwan^3, 4^. Clinical manifestations of CHB can range from asymptomatic to severe chronic liver disease, such as cirrhosis and hepatocellular carcinoma (HCC)^5, 6^. Nucleos(t)ide analogues (NAs) are very effective in treating CHB and viral suppression. However, virological relapse is common after the cessation of NAs, and long-term treatment is required^7–10^. It remains controversial whether or when NAs can be discontinued.

Previous studies showed that hepatitis B surface antigen (HBsAg) quantification has been shown to correlate with HBV DNA and intrahepatic covalently closed circular DNA (cccDNA) levels^11, 12^. However, some studies found that serum HBsAg levels were weakly or moderately correlated with serum HBV DNA, and were weakly or no correlated with intrahepatic cccDNA^13, 14^. Low HBsAg can also predict subsequent HBsAg loss and stratify the risk of HCC^15, 16^. Previous studies demonstrated that serum HBsAg < 100 IU at the end of treatment was a predictor of sustained response after discontinuing NAs^17, 18^. Our previous studies also showed that end-of-treatment HBsAg levels <200 or <150 IU/mL were useful predictors for HBV relapse in HBeAg-negative patients after stopping lamivudine and entecavir treatment, respectively^19, 20^. However, patients with such low HBsAg levels still have a chance of HBV relapse.

Our recent study showed a virological relapse rate of about 25% at 36 months after cessation of entecavir therapy in HBeAg-negative patients who achieved end-of-treatment HBsAg < 150 IU/mL^20^. Thus, further studies are needed to find predictors associated with a high rate of HBV relapse after discontinuing NA therapy in HBeAg-negative patients with low HBsAg levels. The aim of this study is therefore to investigate the rates and predictors of off-therapy HBV relapse and HBsAg loss in HBeAg-negative patients with end-of-treatment HBsAg level ≤200 U/L.

## Results

### Baseline characteristics and HBsAg Levels of the study population

The 119 patient included 95 men and 25 women with a mean age of 52.0 ± 11.7 years at entry. Table 1 shows the clinical characteristics.

**Table 1:** Clinical characteristics of study population.

	Total n = 119
Age (years)	52.0 ± 11.7
Sex (male: female)	94:25
Cirrhosis	28 (23.5%)
FIB-4	4.5 ± 5.1
ALT (U/L) (median, range)	160 (14–3079)
Total bilirubin (mg/dL) (median, range)	1.3 (0.3–24.7)
HBV DNA (log IU/mL)	4.9 ± 1.8
HBV genotype	
B	106 (89.1%)
C	13 (10.9%)
Treatment duration (range) (weeks)	151.5 ± 60.4 (76–346)
Consolidation duration (range) (weeks)	122.9 ± 61.8 (24–322)
HBsAg at baseline (log IU/mL)	2.5 ± 0.9
HBsAg at month 12 of treatment (log IU/mL)	1.9 ± 0.7
HBsAg at the end of treatment (log IU/mL)	1.6 ± 0.8

### Incidence and predictors of post-treatment virological relapse

Among the 119 patients, 45 experienced virological relapse during the follow-up period. The cumulative rates of virological relapse at 12, 36, and 60 months were 25.2%, 38%, and 39.4%, respectively. Table 2 summarizes the risk factors that were predictive of virological relapse. The Cox regression analysis revealed that higher baseline HBV DNA and end-of-treatment HBsAg levels were independent predictors for virological relapse.

**Table 2:** Factors predictive of virological relapse.

Variables	Univariate analysis		Multivariate analysis	
	HR (95% CI)	*p* value	HR (95% CI)	*p* value
Age (per year)	1.022 (0.995–1.049)	0.11		
Sex (Male vs. female)	0.90 (0.44–1.81)	0.76		
Cirrhosis	0.48 (0.20–1.12)	0.09		
FIB-4	0.99 (0.92–1.05)	0.69		
ETV vs. LAM	2.13 (0.99–4.59)	0.053		
ALT (per ULN)	1.000 (1.000–1.001)	0.36		
Total bilirubin (per mg/dL)	1.001 (0.936–1.071)	0.97		
HBV DNA (per log IU/mL)	1.44 (1.21–1.71)	<0.001	1.46 (1.21–1.76)	<0.001
HBV genotype (C vs. B)	0.59 (0.18–1.90)	0.37		
HBsAg at baseline (per log IU/L)	1.46 (1.08–1.98)	0.015		
HBsAg at month 12 of treatment (per log IU/L)	2.53 (1.43–4.46)	0.001		
HBsAg at the end of treatment (per log IU/L)	5.26 (2.22–12.47)	<0.001	5.67 (2.35–13.66)	<0.001
HBsAg decline from baseline to end of treatment (per log IU/L)	0.87 (0.65–1.17)	0.36		
Treatment duration (per week)	1.005 (1.000–1.009)	0.034		
Consolidation duration (per week)	1.004 (0.999–1.008)	0.089		

ALT: alanine aminotransferase, ETV: entecaivr, LAM: lamivudine, HR: hazard ratio, CI: confidence interval.

The end-of-treatment HBsAg level was an independent factor for virological relapse. An HBsAg level of 42 IU/mL was the best cut-off value of for predicting virological relapse within 5 years (AUROC: 0.707). Thus, we used HBsAg level of 50 IU/mL as the optimal value. In patients who had HBV DNA < 50 and ≥50 IU/mL, the virological relapse rates at 60 months were 12.1% and 55.8% (*P* < 0.001), respectively.

HBV DNA levels at entry were also independent predictors of virological relapse. An HBV DNA level of 2 × 10^5^ IU/mL was the best cut-off value of for predicting virological relapse within 5 years (AUROC: 0.825). Thus, we used this level as the optimal value. In patients who had HBV DNA< 2 × 10^5^ and ≥2 × 10^5^ IU/mL, the virological relapse rates at 60 months were 22.2% and 61.3% (*P* < 0.001), respectively.

**Figure 1:**
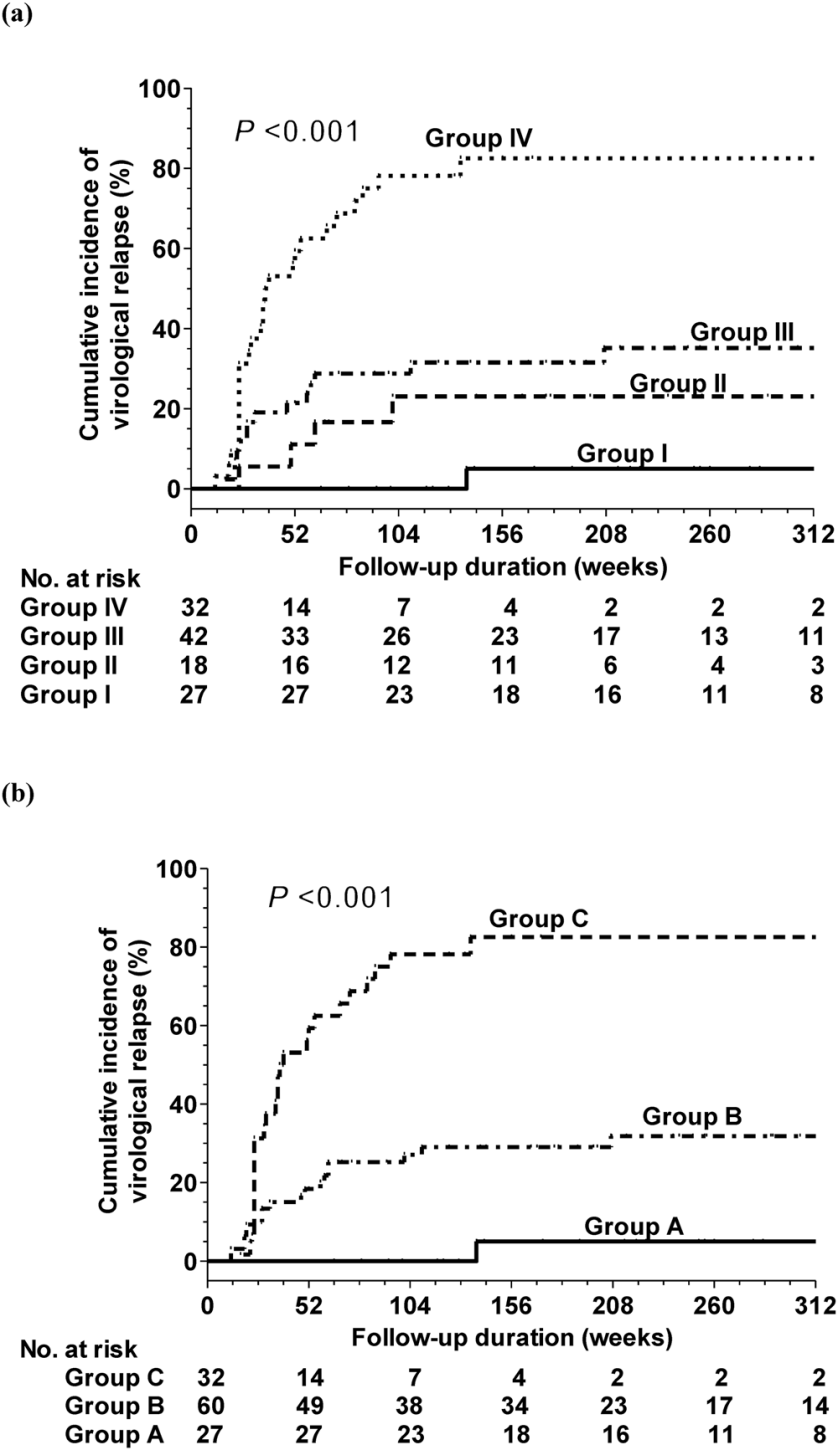
Cumulative incidence of virological relapse according to combining HBV DNA at entry and end-of-treatment HBsAg levels (**a**) Group I: patients with HBV DNA < 2 × 10^5^ IU/mL and HBsAg < 50 IU/mL; Group II: patients with HBV DNA ≥ 2 × 10^5^ IU/mL and HBsAg < 50 IU/mL; Group III: patients with HBV DNA < 2 × 10^5^ IU/mL and HBsAg ≥ 50 IU/mL; and Group IV: patients with HBV DNA ≥ 2 × 10^5^ IU/mL and HBsAg ≥ 50 IU/mL. (**b**) Group A: HBV DNA < 2 × 10^5^ IU/mL and HBsAg < 50 IU/mL; Group B: patients with HBV DNA ≥ 2 × 10^5^ IU/mL and HBsAg < 50 IU/mL or patients with HBV DNA < 2 × 10^5^ IU/mL and HBsAg ≥ 50 IU/mL; and Group C: patients with HBV DNA ≥ 2 × 10^5 ^IU/mL and HBsAg ≥ 50 IU/mL).

We further combined HBV DNA of 2 × 10^5^ IU/mL at entry and end-of-treatment HBsAg levels of 50 IU/mL to assess the combined risk for developing virological relapse. We divided the patients into four subgroups: Group I: HBV DNA < 2 × 10^5^ IU/mL and HBsAg < 50 IU/mL; Group II: HBV DNA ≥ 2 × 10^5^ IU/mL and HBsAg < 50 IU/mL; Group III: HBV DNA < 2 × 10^5^ IU/mL and HBsAg ≥ 50 IU/mL; and Group IV: HBV DNA ≥ 2 × 10^5^ IU/mL and HBsAg ≥ 50 IU/mL. The virological relapse rates at 60 months in Group I to IV were 5%, 35.1%, 23.1%, and 82.5% (*P* < 0.001), respectively (Fig. 1a). After merging group II and III, three were available: Group A: HBV DNA < 2 × 10^5^ IU/mL and HBsAg < 50 IU/mL; Group B: patients with HBV DNA ≥ 2 × 10^5^ IU/mL and HBsAg < 50 IU/mL or patients with HBV DNA < 2 × 10^5^ IU/mL and HBsAg ≥ 50 IU/mL; and Group C: patients with HBV DNA ≥ 2 × 10^5^ IU/mL and HBsAg ≥ 50 IU/mL. The cumulative rates of virological relapse at 60 months in Groups A, B, and C were 5%, 31.8%, and 82.5%, respectively (*P* < 0.001) (Fig. 1b). These three subgroups could also predict virological relapse in the lamivuine (*P* = 0.018) and entecavir groups (*P* < 0.001).

### Incidence and predictors of post-treatment clinical relapse

Among the 119 patients, 29 experienced clinical relapse during the follow-up period. The cumulative rates of clinical relapse at 12, 36, and 60 months were 12.7%, 23.2%, and 27.6%, respectively. The risk factors shown in Table 2 were analyzed for clinical relapse. Cox regression analysis revealed that higher baseline HBV DNA (HR: 1.74, 95% CI: 1.35–2.25, *P* < 0.001) and end-of-treatment HBsAg levels (HR: 5.48, 95% CI: 1.79–16.81, *P* = 0.003) are independent predictors for developing clinical relapse. Of the 45 patients with virological relapse, 14 experienced virological and clinical relapse at the same time, 16 did not experience clinical relapse after virological relapse at the last follow-up, and 15 experienced clinical relapse after virological relapse (the period between virological and clinical relapse: median 43 weeks, range 8–250 weeks).

The end-of-treatment HBsAg and HBV DNA levels at entry were independent predictors of clinical relapse. An HBsAg level of 42 IU/mL was the best cut-off value of for predicting clinical relapse within 5 years (AUROC: 0.705). Thus, we used HBsAg level of 50 IU/mL as the optimal value. In patients who had HBV DNA < 50 and ≥50 log IU/mL, the virological relapse rates at 60 months were 5.4% and 41% (*P* < 0.001), respectively.

The best cut-off value of HBV DNA for predicting clinical relapse within 5 years was 2 × 10^5^ IU/mL (AUROC: 0.864), which we therefore used as the optimal value. The clinical relapse rates at 60 months in patients who had HBV DNA < 2 × 10^5^ and ≥2 × 10^5^ IU/mL were 11.4% and 51.2% (*P* < 0.001), respectively.

**Figure 2:**
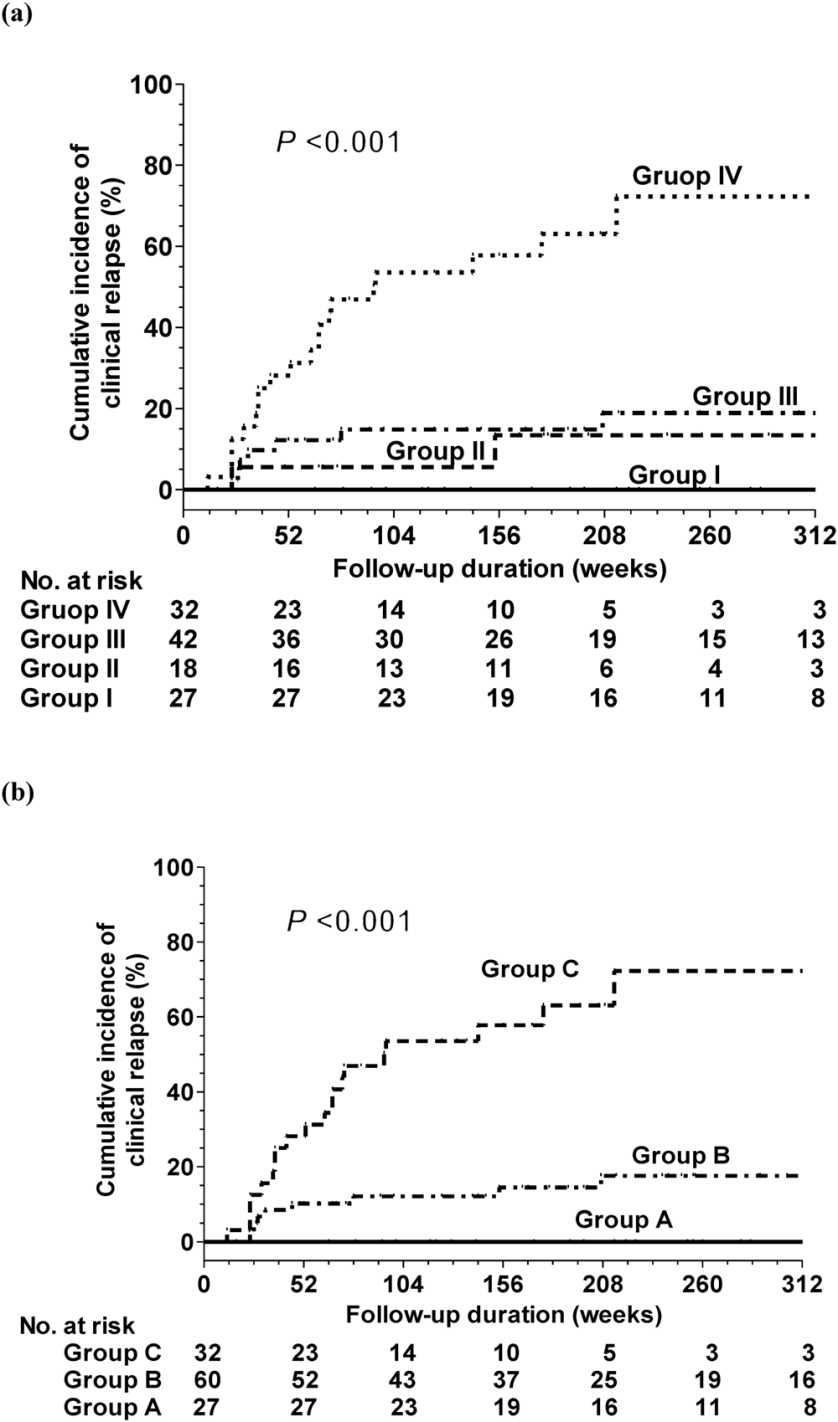
Cumulative incidence of clinical relapse according to combining HBV DNA at entry and end-of-treatment HBsAg levels (**a**) the definition of Group I to IV: as mention Fig. 1a (**b**) the definition of Group A to C: as mention Fig. 1b.

We again combined HBV DNA of 2 × 10^5^ IU/mL at entry and end-of-treatment HBsAg levels of 50 IU/mL into four subgroups as mentioned (Group I to IV). The clinical relapse rates at 60 months in Group I to IV were 0%, 18.9%, 13.4%, and 72.3% (*P* < 0.001), respectively (Fig. 2a). After merging group II and III, three groups as mention above (Group A, B, and C) were available. The cumulative rates of clinical relapse at 60 months in Groups A, B, and C were 0%, 17.4%, and 72.3%, respectively (*P* < 0.001) (Fig. 2b). These three subgroups could also predict clinical relapse in the lamivuine (*P* = 0.012) and entecavir groups (*P* < 0.001).

#### The role of HBsAg levels at 6 months after cessation of entecavir and lamivudine treatment

The HBsAg levels at 6 months after cessation of entecavir and lamivudine treatment were a significant factor for predicting virological relapse (HR: 3.92, 95% CI: 2.33–6.59, *P* < 0.001), clinical relapse (HR: 4.53, 95% CI: 2.29–8.97, *P* < 0.001), and HBsAg loss (HR: 0.46, 95% CI: 0.36–0.58, *P* < 0.001). Compared to increased HBsAg levels, any decline from end-of-treatment HBsAg levels at 6 months post-treatment correlated with significantly lower rates of virological relapse (29.8% vs. 72.3% at 60 months, *P* < 0.001), clinical relapse (20.3% vs. 55.5% at 60 months, *P* = 0.001), and HBsAg loss (52.7% vs. 12.7% at 60 months, *P* = 0.007). In group A patients, the decline levels of HBsAg from end of treatment to 6 months post-treatment was not associated with HBV relapse due to very low rates of virological and clinical relapse. In Group B and C patients, patients who had any decline of HBsAg levels from end of treatment to 6 months post-treatment had a significantly lower rates of virological relapse (38.8% vs. 73.9% at 36 months, *P* < 0.001) and clinical relapse (26.8% vs. 56.2% at 36 months, *P* < 0.001) than those with increased HBsAg levels.

### Incidence and predictors of post-treatment HBsAg loss and hepatic decompensation

**Figure 3:**
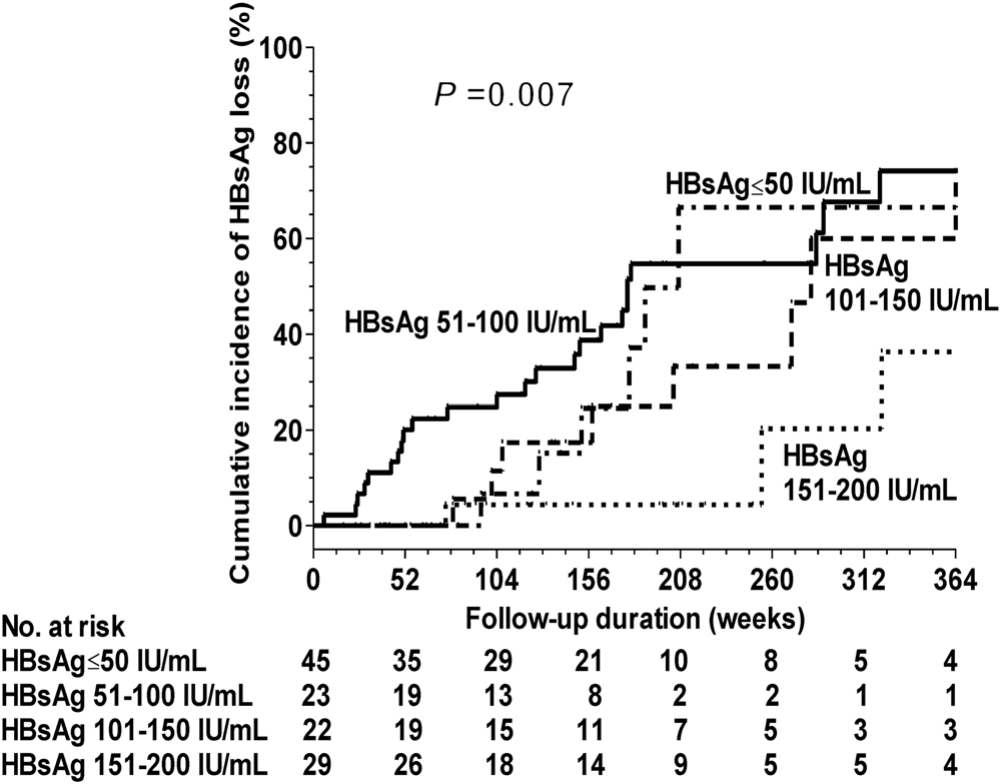
Cumulative incidence of HBsAg loss according to end-of-treatment HBsAg levels.

Among the 119 patients, 44 experienced HBsAg loss during the follow-up period. The cumulative rates of HBsAg loss at 12, 36, and 72 months were 8%, 25.8%, and 54.9%, respectively (the duration of re-treatment was not included). Table 3 summarizes the predictive risk factors for HBsAg loss. The Cox regression analysis revealed that HBV genotype C and end-of-treatment HBsAg levels were independent predictors for the development of HBsAg loss. The cumulative rates of HBsAg loss at 60 months in patients with HBV genotype B and C infections were 40.8% and 63.5%, respectively (*P* = 0.019). The cumulative rates of HBsAg loss in patients with HBsAg ≤ 50 IU/mL, 51–100 IU/mL, 101–150 IU/mL, and 151–200 IU/mL at 60 months were 54.7%, 66.5%, 33.2%, and 20.3%, respectively (Fig. 3). Of the 74 patients without virological relapse, the 6-year cumulative rate of HBsAg loss was 61.2%. Among the 44 patients with HBsAg loss, 9 experienced transient virologial relapse and 2 experienced clinical relapse before HBsAg loss. Retreatment after NA therapy discontinuation was needed for 29 of the 119 patients. No patients experienced ALT flares with hepatic decompensation (bilirubin ≥3 mg/ml and prolonged prothrombin time ≥3 seconds) when clinical relapse occurred after timely retreatment.

**Table 3:** Factors predictive of HBsAg loss.

Variables	Univariate analysis		Multivariate analysis	
	HR (95% CI)	*p* value	HR (95% CI)	*p* value
Age (per year)	0.979 (0.953–1.006)	0.12		
Sex (Male vs. female)	2.12 (0.89–5.03)	0.090		
Cirrhosis	1.71 (0.91–3.23)	0.097		
FIB-4	0.96 (0.88–1.05)	0.39		
ETV vs. LAM	1.01 (0.52–1.98)	0.97		
ALT (per ULN)	1.000 (0.999–1.000)	0.40		
Total bilirubin (per mg/dL)	0.94 (0.87–1.01)	0.10		
HBV DNA (per log IU/mL)	0.97 (0.81–1.17)	0.77		
HBV genotype (C vs. B)	2.46 (1.13–5.37)	0.024	2.55 (1.15–5.62)	0.021
HBsAg at baseline (per log IU/L)	0.88 (0.61–1.27)	0.49		
HBsAg at month 12 of treatment (per log IU/L)	0.43 (0.29–0.65)	<0.001		
HBsAg at the end of treatment (per log IU/L)	0.34 (0.24–0.49)	<0.001	0.33 (0.23–0.47)	<0.001
HBsAg decline from baseline to end of treatment (per log IU/L)	1.83 (1.34–2.51)	<0.001		
Treatment duration (per week)	1.004 (0.999–1.010)	0.14		
Consolidation duration (per week)	1.005 (0.999–1.010)	0.094		

ALT: alanine aminotransferase, HR: hazard ratio, CI: confidence interval.

## Discussion

We found that the rates of virological and clinical relapse at 60 months were 39.4% and 27.6%, respectively, after cessation of lamivudine or entecavir treatment in HBeAg-negative patients who achieved end-of-treatment HBsAg ≤ 200 IU/mL. The Cox regression analysis revealed that higher baseline HBV DNA and end-of-treatment HBsAg levels were independent predictors for virological and clinical relapse. A recent systemic review study showed that the pooled rates of virological relapse at 36 months were 69.9% after NA discontinuation in initially HBeAg-negative patients^21^. Thus, the rate of HBV relapse was not as high in our patients with lower end-of-treatment HBsAg levels.

Our recent studies showed that in HBeAg-negative patients, HBsAg values of 200 and 150 IU/mL could predict sustained responses after lamivudine and entecavir treatment, respectively^19, 20^. A recent study also recognized HBsAg levels <100 IU/ml as a new therapeutic end point^22^. However, we found that the virological relapse rates in patients with end-of-treatment HBsAg ≥ 50 IU/mL were still high (55.8% at 60 months). In contrast, the virological relapse rate in patients who achieved end-of-treatment HBsAg < 50 IU/mL was relatively low (12.1% at 60 months).

For the analysis of clinical relapse, we also found that the virological relapse rates in patients with end-of-treatment HBsAg ≥ 50 IU/mL were still high (40.1% at 60 months). In contrast, the clinical relapse rate in patients with end-of-treatment HBsAg < 50 IU/mL was very low (5.4% at 60 months). Thus, the end-of-treatment HBsAg < 50 IU/mL was more useful for predicting HBV relapse after cessation of entecavir and lamivudine treatment in HBeAg-negative patients.

A previous study showed that HBV DNA ≤ 2 × 10^5^ IU/mL at entry was a significant independent factor for sustained response after cessation of entecavir therapy in HBeAg-negative patients^9^, which our results support. Furthermore, we combined HBV DNA of 2 × 10^5^ IU/mL at entry and end-of-treatment HBsAg to assess the combined risk of virological and clinical relapse and found that the virological and clinical relapse rates were very low (5% and 0% at 60 months, respectively) in patients with a combination of HBsAg < 50 IU/mL and HBV DNA < 2 × 10^5^ IU/mL. In contrast, the virological and clinical relapse rates were high (82.5% and 72.3% at 60 months, respectively) in patients with a combination of HBsAg ≥ 50 IU/mL and HBV DNA ≥ 2 × 10^5^ IU/mL. Thus, close monitoring is needed after the cessation of entecavir and lamivudine therapy in HBeAg-negative patients with a combination of end-of-treatment HBsAg ≥ 50 IU/mL and HBV DNA ≥ 2 × 10^5^ IU/mL at entry.

Investigations of the role of post-treatment HBsAg levels in HBV relapse after cessation of NA therapy were rare. A recent study showed that the HBsAg levels were higher in cases of relapse at 6 months off therapy than among non-relapse cases^22^. Our study also found that the HBsAg level at 6 months post-therapy was a significant factor for predicting virological relapse, clinical relapse, and HBsAg loss. Furthermore, patients who had any decline of HBsAg levels from end of treatment to 6 months post-treatment had a lower virological and clinical relapse rates in high-risk groups than those with increased HBsAg levels. Thus, it is worthwhile to check HBsAg levels at 3 to 6 months after cessation of therapy for early diagnosis of HBV flare, especially in high-risk groups for HBV relapse.

Despite the high HBV relapse rate, we found a relatively high rate of HBsAg loss after cessation of NA therapy in HBeAg-negative patients who achieved end-of-treatment HBsAg ≤ 200 IU/mL. The 6-year cumulative rate of HBsAg loss was 54.9% in these patients. A previous study of HBeAg-negative patients reported a high rate of HBsAg loss of 39% within 6 years after adefovir therapy discontinuation, and 13 (72%) out of 18 patients who achieved a sustained response after stopping adefovir therapy were clear of HBsAg^23^. A recent study found that 9% of the HBeAg-positive patients and 14% of HBeAg-negative patients became HBsAg-negative at 3 years after NA discontinuation^24^. Our study is consistent with these studies^23, 24^. We also found that patients with HBsAg ≤ 50 IU/mL, 51–100 IU/mL, and 101–150 IU/mL had similar rates of HBsAg loss (all more than or near 60% at 72 months). Patients with HBsAg of 151–200 IU/mL had a relatively low rate of HBsAg (20.3% at 72 months). Furthermore, the 6-year cumulative rate of HBsAg loss was 61.2% in 74 patients without virological relapse. NAs have potent antiviral activity but do not have a direct immunomodulatory effect. Thus, HBsAg seroclearance is rare during NA treatment even with the most potent NA^24–27^. The host immune mechanisms that are modulated during the long period of HBV suppression by NA treatment react effectively to the resumed HBV replication. However, for such an immune clearance to occur, the immune system needs not only to become modulated but also to be re-exposed to the antigens of the replicating virus. Thus, stopping NA might more often lead to HBsAg loss than maintaining NA therapy.

In addition to HBsAg, we found that HBV genotype C was another independent factor for predicting off-therapy HBsAg loss in these patients. A recent study in Taiwan showed that compared to genotype B, genotype C infection is associated with a higher chance of spontaneous HBsAg loss in HBeAg-negative patients^28^. However, the mechanism of the impact of genotype C infection on HBsAg loss in HBeAg-negative patients remains unclear.

Most of the current practice guidelines recommend continuing NAs until the loss of HBsAg because of the risk of hepatitis flares and failure. However, recent studies have shown that NA therapy can be safely stopped, even in cirrhotic patients, if proper off-therapy monitoring is provided to restart therapy in a timely manner^8, 9, 29^. But a recent study showed that 2 of 27 patients with cirrhosis developed hepatic decompensation when off NA therapy^22^. In our study, none of patient experienced hepatic decompensation during follow-up or timely retreatment, even among cirrhotic patients. Thus, NA therapy might be safely stopped if proper off-therapy monitoring is provided to restart therapy in a timely manner in HBeAg-negative patients who achieve end-of-treatment HBsAg ≤ 200 IU/mL.

Our study has a few limitations. First, the case number is limited, so further large-scale studies are needed to confirm the results. Second, we only included Asian populations, in which HBV genotypes B and C are predominant^30^, as well as patients who discontinued entecavir and lamivudine treatment. Thus, it is unclear whether the same HBsAg and HBV DNA cut-offs are applicable to other HBV genotypes or for those who have discontinued tenofovir therapy.

In conclusion, after cessation of lamivudine or entecavir treatment in HBeAg-negative patients who achieved end-of-treatment HBsAg ≤ 200 IU/mL, the 5-year rates of virological and clinical relapse and HBsAg loss at 60 months were 39.4%, 27.6%, and 45.9%, respectively. Although the HBV relapse rate was not very high in these patients, patients with a combination of end-of-treatment HBsAg < 50 IU/mL and HBV DNA < 2 × 10^5^ IU/mL at entry experienced the lowest rate of HBV relapse, and this was a better marker for predicting HBV relapse. Patients with a combination of HBV DNA ≥ 2 × 10^5^ IU/mL at entry and end-of-treatment HBsAg ≥ 50 IU/mL had a high HBV relapse rate, and close monitoring is suggested. Patients who achieved end-of-treatment HBsAg ≤ 150 IU/mL had a high rate of HBsAg loss during long-term follow-up. NA therapy might be safely stopped if proper off-therapy monitoring is provided to restart therapy in a timely manner in HBeAg-negative patients who achieve end-of-treatment HBsAg ≤ 200 IU/mL.

## Patients and Methods

### Patients

From 2002 to 2007, 116 HBeAg-negative NA-naïve CHB patients who received lamivudine treatment and had stopped lamivudine for at least 12 months were considered for analysis. All patients fulfilled the antiviral agent stopping criteria according to our previous study^19^. From 2007 to 2011, 240 HBeAg-negative CHB patients who received entecavir treatment and had stopped for at least 12 months were eligible for the analysis. All patients fulfilled the antiviral agent stopping criteria according to the Asian Pacific Association for the Study of the Liver (APASL) 2012 guidelines^31^. Taiwan’s National Health Plan reimbursed CHB patients for oral antiviral agents for 18 months before November 2009 and for 36 months since then. Thus, these patients discontinued NA therapy if they did not want to bear the expenses themselves. Patients were excluded if there was any evidence of autoimmune hepatitis, alcoholic liver disease, markers of hepatitis C virus (HCV), hepatitis D virus, or human immunodeficiency virus (HIV). Patients were also excluded if they lost HBsAg during treatment or had decompensated liver cirrhosis, HCC, or immunosuppressive therapy. Of the 356 HBeAg-negative patients, 119 patients (28 cirrhotic patients) who achieved end-of-treatment HBsAg **≤** 200 U/L were enrolled in this study (34 lamivudine (treatment duration: 95.8 ± 45.7 weeks), 85 entecavir (treatment duration: 174.2 ± 51 weeks)).

Liver biopsy was performed in 14 patients before treatment. None of 14 patients showed cirrhosis. Cirrhosis was diagnosed according to repeated ultrasounds with consistent findings suggestive of cirrhosis in addition to clinical features such as splenomegaly, thrombocytopenia, ascites, or gastroesophageal varices. FIB-4, a simple noninvasive test for liver fibrosis, was defined by the following formula: age (years) × AST(U/L)/{platelets (10^9^/L) × [ALT (U/L)]^1/2^}, where AST represent aspartate aminotransferase^32^.

Post-treatment virological relapse was defined as a serum HBV DNA level > 2000 IU/mL in two consecutive measurements obtained at least 3 months apart after the cessation of NA treatment^19, 20, 23^. Clinical relapse was defined as an episode of alanine aminotransferase (ALT) elevation >2× the upper limit of normal (ULN) (40 U/L) plus HBV DNA > 2000 IU/mL after stopping entecavir treatment^31^. Consolidation duration was calculated from the first demonstration of undetectable HBV DNA to the end of treatment. The criteria of retreatment were HBV DNA ≥ 2000 IU/mL with ALT > 2× ULN in non-cirrhotic patients and whenever HBV DNA ≥ 2000 IU/mL was detected regardless of ALT level in cirrhotic patients.

The study was conducted in accordance with the Helsinki Declaration of 1975. The study was approved by the Ethical Committee of the Chang Gung Memorial Hospital (103-7677B). All patients provided written informed consent.

## Methods

After cessation of lamviudine and entecavir therapy, the patients were followed up every 1 to 3 months. Additional biweekly or weekly visits were arranged if ALT levels increased by more than 2× ULN. The follow-up studies included liver biochemical tests and serological hepatitis B markers. HBV DNA was monitored every three months in the first 12 months and then every 6 months thereafter. HBV DNA was further assessed when clinical relapse was found. If HBV DNA increased beyond 2000 IU/mL during follow-up, HBV DNA was retested 3 months later for confirmation. Ultrasonography was performed for HCC surveillance every 3 to 6 months. Serum HBsAg quantification was performed at baseline, 12 months, the end of treatment during NA therapy, and 6 months after cessation of NA therapy. All serum HBsAg levels were quantified retrospectively using stored serum samples, which had been frozen at −20 °C until use.

### Serology

The presence of HBsAg, HBeAg, anti-HCV antibodies, and anti-HDV antibodies were determined using commercial assay kits (HBsAg EIA, Abbott, North Chicago, IL; HBeAg EIA, Abbott; anti-HCV, EIA 3.0, Abbott; anti-HDV, EIA, Abbott). HBsAg titers were quantified with ElecsysHBsAg II Quant reagent kits (Roche Diagnostics, Indianapolis, IN) according to the manufacturer’s instructions with a lower limit of detection of 0.05 IU/ml. HBV DNA was quantified using the COBAS TaqMan HBV test (CAP-CTM; Roche Molecular Systems, Inc., Branchburg, NJ, USA) with a lower limit of detection of 20 IU/ml.

### HBV genotyping

The HBV genotypes were determined using restriction fragment length polymorphism on the surface gene (between nucleotide positions 256 and 796), as described previously^30^.

### Statistical analysis

Data are presented as the mean ± standard deviation (SD), proportion, or median (range). To compare values between the two groups, the chi-square or Fisher’s exact tests were applied to analyze categorical variables, while the Student’s *t* test was used for continuous variables. The cumulative incidences of post-treatment virological and clinical relapse as well as HBsAg loss were analyzed using the Kaplan-Meier method with a log-rank test. Univariate and multivariate analyses were performed using Cox proportional hazards regression models. A time-dependent receiver-operating characteristic (ROC) curve analysis was used to assess the best HBsAg cut-off level for predicting virological and clinical relapse within 5 years^33^. All statistical tests were two-sided with the significance level set to 0.05.
